# Concurrent neoadjuvant chemoradiotherapy could improve survival outcomes for patients with esophageal cancer: a meta-analysis based on random clinical trials

**DOI:** 10.18632/oncotarget.14669

**Published:** 2017-01-15

**Authors:** Baoxing Liu, Yacong Bo, Kunlun Wang, Yang Liu, Xiance Tang, Yan Zhao, Erjiang Zhao, Ling Yuan

**Affiliations:** ^1^ Affiliated Tumor Hospital of Zhengzhou University, Henan Tumor Hospital, Zhengzhou, Henan, China; ^2^ Department of Nutrition and Food Hygiene, College of Public Health, Zhengzhou University, Affiliated Tumor Hospital of Zhengzhou University, Henan Tumor Hospital, Zhengzhou, China

**Keywords:** neoadjuvant concurrent chemoradiotherapy, esophageal cancer, overall survival, R0 resection rate, progression-free survival

## Abstract

**Background:**

The long-term survival benefit of concurrent neoadjuvant chemoradiotherapy in patients with resectable esophageal cancer remains controversial. In the present study, we conducted a meta-analysis to assess these effectiveness.

**Methods:**

We searched for most relevant studies published up to the end of August 2016, using Pubmed and web of knowledge. And additional articles were identified from previous meta-analysis. The hazard ratio (HR, for overall survival and progression free survival) or risk ratio (RR, for R0 resection) with its corresponding 95 % confidence interval (CI) were used to assess the pooled effect.

**Results:**

Twelve articles including 1756 patients were included in the meta-analysis. Concurrent neoadjuvant chemoradiotherapy followed by surgery was associated with significantly improved overall survival (HR=0.76 , 95% CI= 0.68-0.86), progression survival (HR =0.69, 95% CI= 0.59-0.81), and R0 resection rate(RR =1.17, 95% CI= 1.03-1.33). Subgroup analysis suggested that concurrent neoadjuvant chemoradiotherapy could improve overall survival outcome for squamous cell carcinoma (HR=0.73, 95%CI=0.61-0.88) but not those for adenocarcinoma (HR=0.72, 95%CI=0.48-1.04).

**Conclusion:**

Our findings suggested that concurrent neoadjuvant chemoradiotherapy was associated with a significant survival benefit in patients with esophageal cancer.

## INTRODUCTION

With more than 456,000 newly diagnosed cases and 400,000 related deaths annually, esophageal cancer is the tenth most common cancer and the eighth leading cause of cancer-related deaths worldwide. [1, 2] Since most esophageal cancer patients are diagnosed at the advanced stages, the 5-year survival rate is less than 20%. [3] Despite surgical care and improvements in preoperative staging, surgery alone leads to relatively few long-term survivors. [4] A great number of patients who underwent esophagectomy continue to die as a result of tumor recurrence. [5]

Adjuvant therapies, with either radiotherapy or chemotherapy, have not shown survival any benefits. [5] This, along with the evident difficulties of administering radiotherapy and chemotherapy after resection for esophageal cancer, makes recent trials focus on the role of neoadjuvant treatment, especially the concurrent neoadjuvant chemoradiotherapy (NCRT). A recent meta-analysis showed that concurrent NCRT was associated with improved 1-, 3- and 5-year survival rate. [6] However, the outcomes of their study are pooled risk ratio (RR), which did not consider the survival time, Moreover, analysis for R0 resection and progression-free survival were not conducted.

Several Randomized clinical trials (RCTs) have investigated the effect of concurrent NCRT on operable esophageal cancer. However, these results are controversial. A meta-analysis pooling current literatures might be helpful for confirming such effect. Therefore, the aim of the current study was to determine whether concurrent NCRT plus surgery is superior to surgery alone for operable esophageal cancer.

## RESULTS

### Literature search and study characteristics

**Figure 1:**
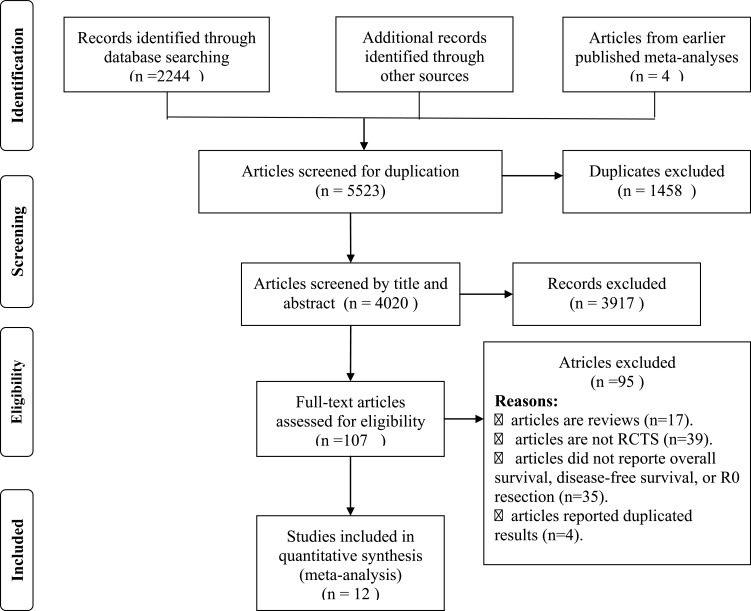
The flow diagram of screened, excluded, and analyzed publications

In total, 12 RCTs including 1756 patients (877patients were treated with concurrent NCRT plus surgery and 879 patients were treated with surgery alone) were included in the meta analysis. [7–18] The detailed processes of our literature search are displayed in Figure 1. Of these 12 studies, twelve studies reported the outcome of overall survival, six studies reported the R0 resection rate, and four studies reported the progression-free survival. The main characters of these studies are presented in Table 1.

**Table 1:** Chemoradiotherapy regimens in randomized trials included in the meta-analysis

First author	Year	Sample size			Pathology	Neoadjuvant treatment schedule
		NCRTS	SA	total		
Wlsh	1996	58	55	113	AC	Cis 75mg/m2 on days 7 and 42; FU 15 mg/kg on days 1-5 and 36-40 40 Gy in 15 fractions over 3weeks
Shapiro	2015	178	188	366	AC and SCC	Carboplatin (AUC 2 mg/mL per min) and paclitaxel (50 mg/m^2^ of body-surface area) were administered intravenously for five cycles, starting on days 1, 8, 15, 22, and 29. A total concurrent radiation dose of 41·4 Gy was given in 23 fractions of 1·8 Gy, on 5 days per week (excluding weekends), starting on the fi rst day of the first chemotherapy cycle.
Mariette	2014	81	89	170	AC and SCC	A total dose of 45 Gy was delivered in 25 fractions (five fractions per week) over 5 weeks. Chemotherapy was delivered concomitantly and composed of two cycles of fluorouracil (FU) and cisplatin. FU 800 mg/m2 per 24 hours was administered as a continuous infusion from days 1 to 4 and 29 to 32. Cisplatin 75mg/m2 was delivered by infusiononday 1 or 2 and againonday 29 or 30.
Lv	2010	80	80	160	SCC	Radiation was delivered in a total dose of 40 Gy (20 fractions at 2 Gy per fraction). For chemotherapy, 2 cycles were administered on days 1-3 and days 22-24 of radiotherapy. A paclitaxel (PTX)+cisplatin (DDP) regimen was used, including PTX (135 mg/m2 per day) administered as a short-term infusion on day 1 of each cycle, while DDP (20 mg/m2 per day) was delivered as a continuous infusion over 24 h on days 1-3 of each cycle.
Burmeister	2005	128	128	256	AC and SCC	Cis 80mg/m2 on day 1; FU 800 mg/m2 per day on days 1-4, 35 Gy in 15 fractions over 3 weeks
Cao	2009	118	118	236	SCC	Cis 20 mg/m2 per day on days 1–5; FU 500 mg/m2 per day on days 1–5; mitomycin 10mg/m2 per day on day 1 40 Gy, 2 Gy per fraction over 4weeks
Lee	2004	51	50	101	SCC	Cis 60 mg/m2 on days 1 and 22; FU 1000 mg/m2 per day on days 2–5. 45·6Gy, 1·2Gy per fraction over 28 days
Tepper	2008	30	26	56	AC and SCC	Cis 60mg/m2 on days 1 and 29; FU 1000 mg/m2 per day on days 1–4 and 29–32 50·4Gy, 1·8Gy per fraction over 5·6weeks
Natsugoe	2006	22	23	45	SCC	A total radiation dose of 40 Gy was applied, in 2-Gy fractions delivered 5 days/week for 4 weeks to the media stinum and neck. In the same period, intravenous chemotherapy was performed using cisplatin (7 mg over 2 h) and 5-fluorouracil (5-FU; 350 mg over 24 h).
Urba	2007	50	50	100	AC and SCC	Cis 20mg/m2 on days 1–5 and 17–21; FU 300 mg/m2 on days 1–21; vinblastine 1 mg/m2 on days 1–4 and 17–20 45 Gy, 1·5Gy per fraction over 3weeks
Hsu	2013	46	38	84	SCC	The chemotherapy regimen included 80 mg/m2 of cisplatin intravenously on day 1 followed by 600 mg/m2/day of 5-fluorouracil and 90 mg/m2/day of leucovorin given by continuous intravenous infusion on days 1–4, concurrent with 45.0–50.4 Gy of externalbeam radiation at 1.8–2.0 Gy per fraction.
Apinop	1994	35	34	69	SCC	Cis 100 mg/m2 on days 1 and 29; FU 1000 mg/m2 per day on days 1–4 and 29–32 40 Gy, 2 Gy per fraction over 4weeks

NCRTS, neoadjuvant chemoradiotherapy followed by surgery; SA , surgery alone; SCC, squamous cell carcinoma; AC, adenocarcinoma; Cis, cisplatin; FU, fluorouracil

### Primary outcome

**Figure 2:**
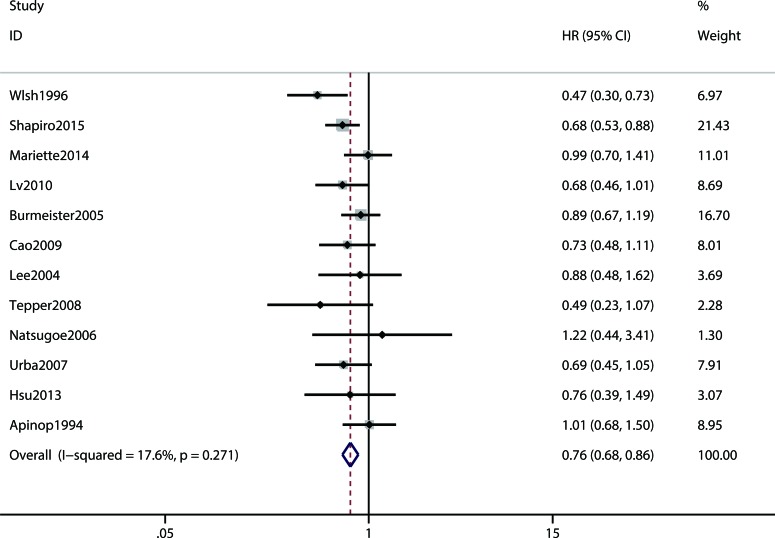
Meta analysis comparing the overall survival between neoadjuvant chemoradiotherapy plus surgery and surgery alone. CI, confidence interval; HR, hazard ratio

The primary outcome overall survival was reported in twelve RCTs. Compared with the surgery alone group, the pooled hazard ratio (HR) for the concurrent NCRT plus surgery group was 0.76 (95% CI 0.68-0.86) (Figure 2). As presented in Table 2, when we carried out the stratified analysis by geographical location, significant results were observed both in the west and east (HR = 0.75, 95%CI = 0.64-0.85 and HR = 0.82, 95%CI = 0.67-0.88, respectively). Furthermore, the subgroup analysis by histological type showed that concurrent NCRT plus surgery can improve squamous cell carcinoma (SCC) patients (HR = 0.73, 95%CI = 0.61-0.88), but not adenocarcinoma (AC) patients (HR = 0.72, 95%CI = 0.48-1.04) or AC+SCC patients (HR = 0.80, 95%CI = 0.62-1.04).

### Secondary outcomes

**Figure 3:**
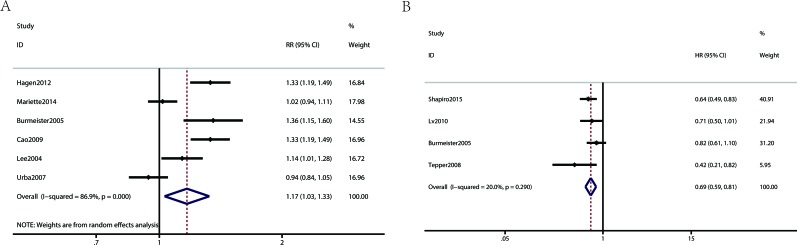
Meta analysis comparing the secondary outcomes of patients receiving neoadjuvant chemoradiotherapy plus surgery and surgery alone (**A**: R0 resection; **B**: progression-free survival).

Figure 3 show Forest plots for the secondary outcomes, including the R0 resection rate and progression-free survival. Four RCTs reported the progression-free survival, indicating a statistically significant difference (HR 0.69, 95% CI 0.59-0.81, Figure 3B) for the concurrent NCRT plus surgery group compared to the surgery alone group. Six RCTs reported the R0 resection rate, indicating a statistically significant difference (RR 1.17, 95% CI 1.03-1.33, Figure 3A) for the concurrent NCRT plus surgery group compared to the surgery alone group.

**Table 2:** Subgroup analysis for overall survival of concurrent NCRT plus surgery vs. surgery alon

Subgroups	Included studies	Sample size		HR (95 % CI)	*P* value for heterogeneity
		NCRTS	SA		
Geographical location					
West	6	525	536	0.74(0.64-0.85)	0.081
East	6	352	343	0.82(0.67-1.00)	0.714
Histology					
SCC	8	438	436	0.76(0.63-0.90)	0.508
AC	3	272	274	0.72(0.48-0.1.08)	0.021
AC+SCC	3	161	165	0.80(0.62-1.04)	0.182

NCRTS, neoadjuvant chemoradiotherapy followed by surgery; SA , surgery alone; SCC, squamous cell carcinoma; AC, adenocarcinoma; HR, hazard ratio; CI: confidence interval.

### Publication bias

**Figure 4:**
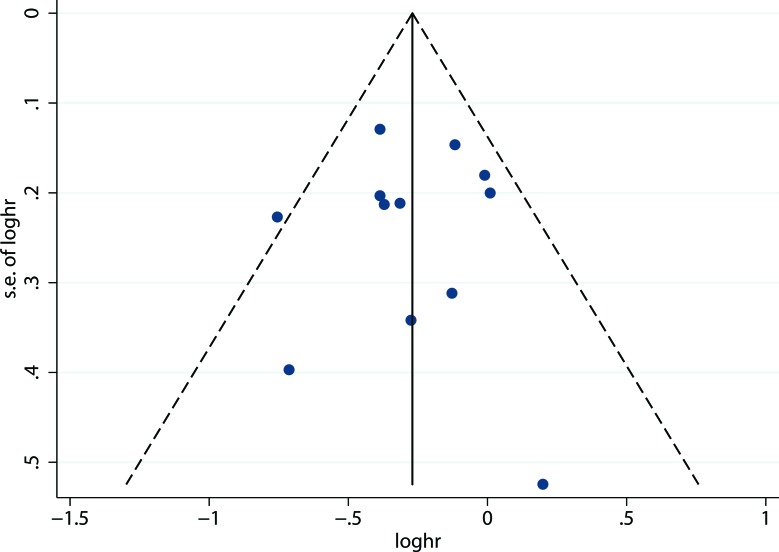
Funnel plot for publication bias of overall survival between neoadjuvant chemoradiotherapy plus surgery and surgery alone

A funnel plot of the effect size for overall survival was found to be symmetrical (Figure. 4), indicating little publication bias. The *P* value based on Egger's test for overall survival was 0.990 (*t* = -0.01), which also showed no publication bias existed.

## DISCUSSION

The results of present meta-analysis showed that concurrent NCRT was associated with improved overall survival, progression-free survival, and R0 resection rate in patients with esophageal cancer. And this is the largest meta-analysis to explore the survival benefit with concurrent NCRT plus surgery *versus* surgery alone.

Several potential explanations exist for the improved effects of concurrent NCRT. First, chemotherapy has a radio sensitizing effect, which can enhance the local effects of concurrent radiotherapy, reducing the possibility of tumor spreading from the primary tumor site prior to surgery. Second, concurrent chemoradiotherapy may have the ability to inhibit the proliferation of tumor cells in the primary lesion site, decreasing the length of preoperative treatment required.

Our subgroup analysis also demonstrated that concurrent NCRT plus surgery significantly improved overall survival outcome for patients in the west. Whereas in the east, concurrent NCRT plus surgery was only associated with improved overall outcome with a borderline significant. It might be ascribed the different genetic backgrounds and/or ethnicities of the participants, which should be further investigated with well-designed multicenter RCTs from different ethnics. Moreover, in order to verify the effect of concurrent NCRT, subgroup analysis based on histology was also introduced. Through this subgroup analysis, we found that concurrent NCRT plus surgery improved the overall survival outcome for SCC patients, but not for AC patients or AC+SCC patients. Several explanations may partly explain the reasons. Firstly, participants with SCC may gain more benefits from concurrent NCRT plus surgery than those with AC. Secondly, SCC patients have a greater potential to involve the superior mediastinum and cervical region than AC patients, which is more difficult for aggressive intrathoracic and cervical nodal dissection to be carried out. Besides, SCC is more sensitive to radiotherapy and chemotherapy than AC. [6] Thus, the addition of chemoradiotherapy to the treatment regime might have improved therapeutic outcome.

In order to determine the reasons that patients with resectable esophageal cancer could receive a survival advantage from concurrent NCRT, we chose the R0 resection rate as a secondary outcome. The final results revealed that the RR of R0 resection rate favored the group of concurrent NCRT, which approximated the results of previous study. [19] However, they included not only concurrent NCRT but also sequential NCRT, suggesting that the combination of concurrent and sequential NCRT rather than concurrent NCRT was the likely cause of improved R0 resection. In addition to R0 resection rate, our meta-analysis also investigated progression-free survival which is rarely published in previous meta-analysis. Compared with surgery alone, concurrent NCRT plus surgery significantly improved the progression-free survival. This finding also demonstrated that concurrent NCRT plus surgery could improve therapeutic outcomes.

Although subgroup analysis based on postoperative complications failed to be conducted, the included studies showed that treatment-related complications in the NCRT group had no significant difference from that of the surgery alone group [13, 17]. In addition, loss to follow-up was low (ranging from 0.4% to 4.2%), thus the result from our study is robust.

Several potential limitations of our meta-analysis should also be acknowledged. At first, since most studies included patients without clear identification of tumor-node-metastasis (TNM) classification, the subgroup based on TNM classification could not be achieved. In addition, as the primary endpoint was overall survival, the difference of complications between NRCT plus surgery group and surgery alone group was not clear.

In conclusion, concurrent NCRT improves overall survival, progression-free survival and R0 resection rate in patients with esophageal cancer. This improvement is statistically significant and clinically relevant for SCC but not for AC subtypes. Concurrent NCRT followed by surgery should be viewed as a standard care for patients with resectable esophageal squamous cell carcinoma. Further well-designed multicenter RCTs are warranted to verify the beneficial effect.

## MATERIALS AND METHODS

### Search strategy

A comprehensive literature search of Pubmed, and Web of Knowledge was conducted up to the end of August 2016. The following search formula were used: (([esophageal ] OR [oesophageal] OR [esophagus] OR [oesophagus]) AND ([neoplasms] OR [cancer] OR [carcinoma]) AND ([chemotherapy] OR [radiotherapy] OR [chemoradiotherapy] OR [combined modality therapy] OR [adjuvant]) AND [neoadjuvant]). The searches were limited to articles describing RCTS and published in English. In order to identify further additional studies, manual searching of reference lists was performed.

### Study selection

Articles included should fit all the following criteria: (1) Studies were designed as RCTs; (2) compared concurrent NCRT with surgery alone were considered; (3) the outcome of interest was defined as overall survival, progression-free survival, or R0 resection rate; and (4) the sample size, hazard ratio (HR) and their 95 % confidence interval (CI), or data that would allow those findings to be inferred, was presented. If several publications reporting on the same population data met our criteria, the one with the longest follow-up period was selected.

### Data extraction and outcome measures

The primary outcome was overall survival. The secondary outcomes were progression-free survival and R0 resection rate, which was defined by a tumor-free resection margin. Two authors (Baoxing Liu and Yacong Bo) independently extracted the following data from each eligible study, and discrepancies were resolved by a third investigator: first author, year of publication, number of patients randomized, and those who received chemoradiotherapy or surgery, HR for overall survival and progression-free survival, the total number of participants for each study, and the number of patients for R0 resection.

### Statistical analysis

The meta-analysis was performed using STATA software (version 12.0; StatCorp, College Station, TX, USA) and *P* < 0.05 was considered as statistically significant. Overall survival and progression-free survival were measured with a hazard ratio (HR), while the R0 resection rate was measured using risk ratios (RR). If permitted, HR and the 95% confidence interval (CI) were obtained directly from the article; otherwise, they were calculated using the methods of Parmar, [20] Tierney, [21] and Williamson, [22] which use number of events and Kaplan-Meier survival curves to estimate the HR and 95% CI. The chi-square test and I^2^ test were used to assess heterogeneity, with *P* < 0.05 and/or *I*^2^ > 50% representing significant heterogeneity, and the random-effect model was selected. Otherwise, a fixed-effect model was applied [23]. Subgroup analyses were applied to evaluate potential effect modification of variables including geographic locations, and histological type (SCC and AC). Begger's Funnel plots and Egger's tests were performed to assess the publication bias [24, 25].
